# Supplementary material for: Inhibition of Aurora Kinase B attenuates fibroblast activation and pulmonary fibrosis
Source: EMBO Mol Med. 2020 Aug 6;12(9):e12131. doi: 10.15252/emmm.202012131 (PMC7507328; doi:10.15252/emmm.202012131)
Supplement: Supplementary file 1 — Appendix [file EMMM-12-e12131-s001.pdf]

Inhibition of Aurora Kinase B Attenuates Fibroblast Activation and Pulmonary Fibrosis

Rajesh K. Kasam[1,2], Sudhir Ghandikota[3,4], Divyalakshmi Soundararajan[1], Geereddy B. Reddy[2], Steven K. Huang[5], Anil G. Jegga[3,4,6,*], and Satish K. Madala[1,6,*]

Appendix

Table of contents:

Appendix Fig S1 (Page 2-3)

Appendix Table S1 (Page 4-8)

Appendix Table S2-S6 (Page 9-13)

Appendix Figure S1.

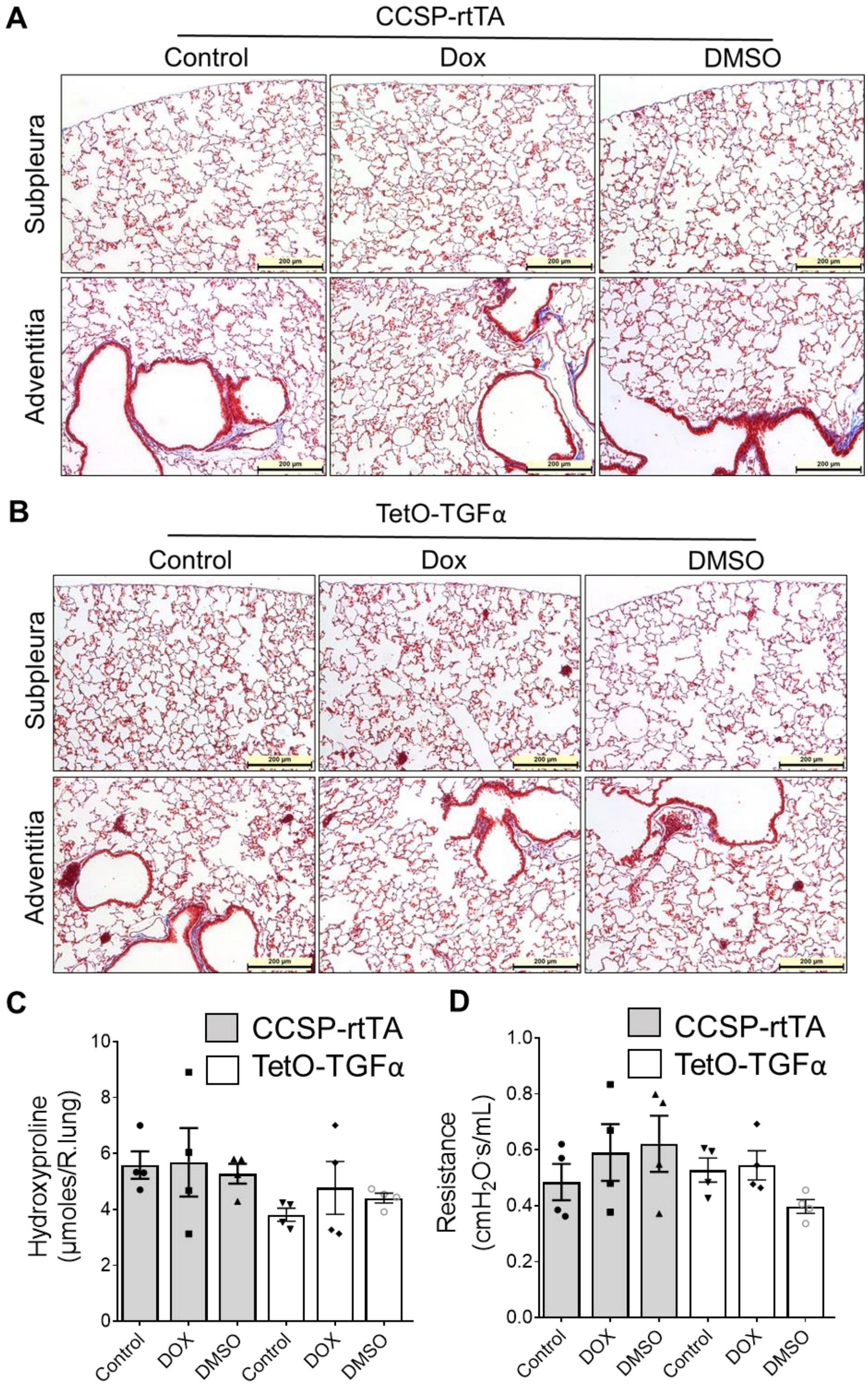

Appendix Fig S1. Doxycycline or DMSO treatment does not induce fibrosis or inflammation

CCSP-rtTA and TetO-TGFα mice were either treated with 5% DMSO for 3 wks or fed with DOX-containing food for 6 wks.

(A) Representative images of Masson's trichrome stained lung sections from CCSP-rtTA mice. Images were obtained at 10X magnification. Scale bar, 200 μm. (n=4 mice/group).

(B) Representative images of Masson's trichrome stained lung sections from TetO-TGFα mice. Images were obtained at 10X magnification. Scale bar, 200 μm. (n=4 mice/group).

(C) Quantification of total lung hydroxyproline levels. (n=4 mice/group).

(D) Resistance was measured using FlexiVent. (n=4 mice/group).

Appendix Table S1. The list of IPF genes that are up or down regulated with the knockdown of AURKB in lung fibroblasts isolated from TGFα mice on Dox for 10 days.

Gene ID	symbol	Annotation	log2 FC	pval	padj (FDR)
17523	*Mpo*	IPF-DN siAURKB-UP	6.35	0	0
12424	*Cck*	IPF-DN siAURKB-UP	3.68	0	0
14058	*F10*	IPF-DN siAURKB-UP	3.48	0	0
20201	*S100a8*	IPF-DN siAURKB-UP	2.81	0	0
20510	*Slc1a1*	IPF-DN siAURKB-UP	2.21	0	0.01
20349	*Sema3e*	IPF-DN siAURKB-UP	1.91	0	0
107869	*Cth*	IPF-DN siAURKB-UP	1.91	0	0
71950	*Nanog*	IPF-DN siAURKB-UP	1.83	0	0
210530	*P3h2*	IPF-DN siAURKB-UP	1.56	0	0
76574	*Mfsd2a*	IPF-DN siAURKB-UP	1.45	0.02	0.05
105349	*Akr1c18*	IPF-DN siAURKB-UP	1.39	0	0
108052	*Slc14a1*	IPF-DN siAURKB-UP	1.39	0	0
230603	*Ttc39a*	IPF-DN siAURKB-UP	1.27	0.01	0.02
18218	*Dusp8*	IPF-DN siAURKB-UP	1.19	0	0
13113	*Cyp3a13*	IPF-DN siAURKB-UP	1.07	0.01	0.02
16169	*Il15ra*	IPF-DN siAURKB-UP	1.01	0	0.01
14226	*Fkbp1b*	IPF-DN siAURKB-UP	1	0	0
57339	*Jph1*	IPF-DN siAURKB-UP	1	0	0
30956	*Aass*	IPF-DN siAURKB-UP	0.98	0	0
15446	*Hpgd*	IPF-DN siAURKB-UP	0.95	0	0
268949	*Dpcr1*	IPF-DN siAURKB-UP	0.94	0	0
56811	*Dkk2*	IPF-DN siAURKB-UP	0.94	0	0
227327	*B3gnt7*	IPF-DN siAURKB-UP	0.86	0	0
23972	*Papss2*	IPF-DN siAURKB-UP	0.84	0	0
236643	*Sytl5*	IPF-DN siAURKB-UP	0.84	0	0
244209	*Cyp2r1*	IPF-DN siAURKB-UP	0.82	0	0
68070	*Pdzd2*	IPF-DN siAURKB-UP	0.8	0	0
74513	*Neto2*	IPF-DN siAURKB-UP	0.78	0	0
243958	*Siglecg*	IPF-DN siAURKB-UP	0.77	0	0
16194	*Il6ra*	IPF-DN siAURKB-UP	0.77	0	0
100342	*Fam46b*	IPF-DN siAURKB-UP	0.76	0	0
22339	*Vegfa*	IPF-DN siAURKB-UP	0.75	0	0
27222	*Atp1a4*	IPF-DN siAURKB-UP	0.74	0.02	0.04
19088	*Prkar2b*	IPF-DN siAURKB-UP	0.73	0	0
12064	*Bdnf*	IPF-DN siAURKB-UP	0.72	0	0
11555	*Adrb2*	IPF-DN siAURKB-UP	0.7	0.01	0.02
70484	*Slc35d2*	IPF-DN siAURKB-UP	0.69	0	0
67606	*Fibin*	IPF-DN siAURKB-UP	0.69	0	0

52626	*Cdkn2aipnl*	IPF-DN siAURKB-UP	0.68	0	0
22439	*Xk*	IPF-DN siAURKB-UP	0.67	0	0
14167	*Fgf12*	IPF-DN siAURKB-UP	0.65	0	0.01
71091	*Cdkl1*	IPF-DN siAURKB-UP	0.64	0	0
83554	*Fstl3*	IPF-DN siAURKB-UP	0.64	0	0
26568	*Slc27a3*	IPF-DN siAURKB-UP	0.64	0	0
84682	*Cox4i2*	IPF-DN siAURKB-UP	0.63	0.01	0.04
16475	*Ajuba*	IPF-DN siAURKB-UP	0.62	0	0
30963	*Hacd1*	IPF-DN siAURKB-UP	0.62	0	0
17756	*Map2*	IPF-DN siAURKB-UP	0.62	0.01	0.02
233733	*Galnt18*	IPF-DN siAURKB-UP	0.6	0.01	0.02
71761	*Amdhd1*	IPF-DN siAURKB-UP	0.59	0	0.02
228026	*Pdk1*	IPF-UP siAURKB-DN	-0.59	0	0
70974	*Pgm2l1*	IPF-UP siAURKB-DN	-0.59	0	0
68867	*Rnf122*	IPF-UP siAURKB-DN	-0.6	0	0
13038	*Ctsk*	IPF-UP siAURKB-DN	-0.61	0	0
12628	*Cfh*	IPF-UP siAURKB-DN	-0.61	0	0
22782	*Slc30a1*	IPF-UP siAURKB-DN	-0.61	0	0
192897	*Itgb4*	IPF-UP siAURKB-DN	-0.61	0	0
14667	*Gm2a*	IPF-UP siAURKB-DN	-0.62	0	0
13078	*Cyp1b1*	IPF-UP siAURKB-DN	-0.62	0	0
12819	*Col15a1*	IPF-UP siAURKB-DN	-0.63	0	0
12494	*Cd38*	IPF-UP siAURKB-DN	-0.63	0	0
75869	*Arl5b*	IPF-UP siAURKB-DN	-0.63	0	0
30878	*Apln*	IPF-UP siAURKB-DN	-0.63	0	0
223753	*Cerk*	IPF-UP siAURKB-DN	-0.64	0	0
211770	*Trib1*	IPF-UP siAURKB-DN	-0.64	0	0
74782	*Glt8d2*	IPF-UP siAURKB-DN	-0.65	0	0
20315	*Cxcl12*	IPF-UP siAURKB-DN	-0.66	0	0
269356	*Slc4a11*	IPF-UP siAURKB-DN	-0.66	0.01	0.02
15465	*Hrh1*	IPF-UP siAURKB-DN	-0.67	0.01	0.02
23893	*Grem2*	IPF-UP siAURKB-DN	-0.67	0	0
213311	*Fbxl21*	IPF-UP siAURKB-DN	-0.67	0.01	0.04
114886	*Cygb*	IPF-UP siAURKB-DN	-0.67	0	0
211550	*Tifa*	IPF-UP siAURKB-DN	-0.68	0	0
22704	*Zfp46*	IPF-UP siAURKB-DN	-0.68	0	0
12870	*Cp*	IPF-UP siAURKB-DN	-0.69	0	0
98952	*Fam102a*	IPF-UP siAURKB-DN	-0.69	0	0
13653	*Egr1*	IPF-UP siAURKB-DN	-0.7	0	0
79221	*Hdac9*	IPF-UP siAURKB-DN	-0.71	0	0.01
22031	*Traf3*	IPF-UP siAURKB-DN	-0.71	0	0

18190	*Nrxn2*	IPF-UP siAURKB-DN	-0.71	0	0
12043	*Bcl2*	IPF-UP siAURKB-DN	-0.71	0	0
17150	*Mfap2*	IPF-UP siAURKB-DN	-0.72	0	0
23796	*Aplnr*	IPF-UP siAURKB-DN	-0.73	0.02	0.05
67405	*Nts*	IPF-UP siAURKB-DN	-0.73	0	0
69538	*Antxr1*	IPF-UP siAURKB-DN	-0.74	0	0
319757	*Smo*	IPF-UP siAURKB-DN	-0.74	0	0
11932	*Atp1b2*	IPF-UP siAURKB-DN	-0.75	0	0
14962	*Cfb*	IPF-UP siAURKB-DN	-0.75	0	0
93890	*Pcdhb19*	IPF-UP siAURKB-DN	-0.75	0	0
29863	*Pde7b*	IPF-UP siAURKB-DN	-0.75	0	0
629059	*Fam124a*	IPF-UP siAURKB-DN	-0.76	0	0
67896	*Ccdc80*	IPF-UP siAURKB-DN	-0.76	0	0
353211	*Prune2*	IPF-UP siAURKB-DN	-0.77	0	0
16181	*Il1rn*	IPF-UP siAURKB-DN	-0.77	0.02	0.05
74645	*Fam46c*	IPF-UP siAURKB-DN	-0.77	0	0
215748	*Cnksr3*	IPF-UP siAURKB-DN	-0.77	0	0
12142	*Prdm1*	IPF-UP siAURKB-DN	-0.78	0.01	0.04
55987	*Cpxm2*	IPF-UP siAURKB-DN	-0.78	0	0
17540	*Mrvi1*	IPF-UP siAURKB-DN	-0.8	0	0
171463	*Il17rd*	IPF-UP siAURKB-DN	-0.81	0	0
26941	*Slc9a3r1*	IPF-UP siAURKB-DN	-0.82	0	0
19737	*Rgs5*	IPF-UP siAURKB-DN	-0.82	0	0
20361	*Sema7a*	IPF-UP siAURKB-DN	-0.82	0	0
93892	*Pcdhb21*	IPF-UP siAURKB-DN	-0.85	0	0
21858	*Timp2*	IPF-UP siAURKB-DN	-0.86	0	0
56847	*Aldh1a3*	IPF-UP siAURKB-DN	-0.87	0.01	0.03
19219	*Ptger4*	IPF-UP siAURKB-DN	-0.87	0	0
80718	*Rab27b*	IPF-UP siAURKB-DN	-0.88	0	0
14579	*Gem*	IPF-UP siAURKB-DN	-0.88	0	0
66695	*Aspn*	IPF-UP siAURKB-DN	-0.89	0	0
11816	*Apoe*	IPF-UP siAURKB-DN	-0.89	0	0
28109	*D10Wsu102e*	IPF-UP siAURKB-DN	-0.89	0	0
320916	*Wscd2*	IPF-UP siAURKB-DN	-0.9	0	0.01
53901	*Rcan2*	IPF-UP siAURKB-DN	-0.9	0	0
22329	*Vcam1*	IPF-UP siAURKB-DN	-0.9	0	0
20897	*Stra6*	IPF-UP siAURKB-DN	-0.91	0	0
16416	*Itgb3*	IPF-UP siAURKB-DN	-0.91	0	0
20429	*Shox2*	IPF-UP siAURKB-DN	-0.92	0.01	0.02
238377	*Gpr68*	IPF-UP siAURKB-DN	-0.93	0	0
14089	*Fap*	IPF-UP siAURKB-DN	-0.95	0	0

50706	*Postn*	IPF-UP siAURKB-DN	-0.97	0	0
12818	*Col14a1*	IPF-UP siAURKB-DN	-0.97	0	0
101772	*Ano1*	IPF-UP siAURKB-DN	-0.98	0	0.01
16324	*Inhbb*	IPF-UP siAURKB-DN	-0.98	0	0
27528	*Nrep*	IPF-UP siAURKB-DN	-0.99	0	0
64074	*Smoc2*	IPF-UP siAURKB-DN	-0.99	0	0
72536	*Tagap*	IPF-UP siAURKB-DN	-1.01	0	0
69707	*Iqcg*	IPF-UP siAURKB-DN	-1.03	0	0
20972	*Syngr1*	IPF-UP siAURKB-DN	-1.03	0	0
16590	*Kit*	IPF-UP siAURKB-DN	-1.03	0	0
14164	*Fgf1*	IPF-UP siAURKB-DN	-1.04	0.01	0.03
230779	*Serinc2*	IPF-UP siAURKB-DN	-1.04	0.01	0.03
107587	*Osr2*	IPF-UP siAURKB-DN	-1.05	0	0
15160	*Serpind1*	IPF-UP siAURKB-DN	-1.08	0	0
243743	*Plxna4*	IPF-UP siAURKB-DN	-1.09	0	0
246316	*Lgi2*	IPF-UP siAURKB-DN	-1.09	0	0
21810	*Tgfbi*	IPF-UP siAURKB-DN	-1.1	0	0
17470	*Cd200*	IPF-UP siAURKB-DN	-1.11	0	0
383619	*Aim2*	IPF-UP siAURKB-DN	-1.11	0	0.01
20394	*Scg5*	IPF-UP siAURKB-DN	-1.11	0	0.02
20257	*Stmn2*	IPF-UP siAURKB-DN	-1.14	0	0
13537	*Dusp2*	IPF-UP siAURKB-DN	-1.15	0.01	0.03
30955	*Pik3cg*	IPF-UP siAURKB-DN	-1.16	0	0.01
54720	*Rcan1*	IPF-UP siAURKB-DN	-1.18	0	0
20284	*Scrg1*	IPF-UP siAURKB-DN	-1.18	0	0.01
242341	*Atp6v0d2*	IPF-UP siAURKB-DN	-1.18	0.01	0.02
56226	*Espn*	IPF-UP siAURKB-DN	-1.21	0	0
67731	*Fbxo32*	IPF-UP siAURKB-DN	-1.22	0	0
12759	*Clu*	IPF-UP siAURKB-DN	-1.22	0	0
15186	*Hdc*	IPF-UP siAURKB-DN	-1.23	0.01	0.04
229898	*Gbp5*	IPF-UP siAURKB-DN	-1.26	0	0
20296	*Ccl2*	IPF-UP siAURKB-DN	-1.28	0	0
16651	*Sspn*	IPF-UP siAURKB-DN	-1.3	0	0
19126	*Prom1*	IPF-UP siAURKB-DN	-1.33	0	0
66797	*Cntnap2*	IPF-UP siAURKB-DN	-1.34	0	0.01
18053	*Ngfr*	IPF-UP siAURKB-DN	-1.36	0	0.01
20452	*St8sia4*	IPF-UP siAURKB-DN	-1.37	0	0
270190	*Ephb1*	IPF-UP siAURKB-DN	-1.37	0	0
96935	*Susd4*	IPF-UP siAURKB-DN	-1.39	0.01	0.04
269642	*Nat8l*	IPF-UP siAURKB-DN	-1.41	0	0
14064	*F2rl2*	IPF-UP siAURKB-DN	-1.44	0.01	0.03

319655	*Podxl2*	IPF-UP siAURKB-DN	-1.45	0	0
20292	*Ccl11*	IPF-UP siAURKB-DN	-1.46	0	0
229933	*Clca2*	IPF-UP siAURKB-DN	-1.48	0	0
20319	*Sfrp2*	IPF-UP siAURKB-DN	-1.48	0	0
11931	*Atp1b1*	IPF-UP siAURKB-DN	-1.51	0	0
56720	*Tdo2*	IPF-UP siAURKB-DN	-1.51	0.01	0.02
270192	*Rab6b*	IPF-UP siAURKB-DN	-1.52	0	0
107753	*Lgals2*	IPF-UP siAURKB-DN	-1.53	0	0
68498	*Tspan11*	IPF-UP siAURKB-DN	-1.58	0	0.01
78249	*Adgrf4*	IPF-UP siAURKB-DN	-1.6	0	0
56788	*Scube2*	IPF-UP siAURKB-DN	-1.65	0	0
54156	*Egfl6*	IPF-UP siAURKB-DN	-1.65	0.01	0.04
18039	*Nefl*	IPF-UP siAURKB-DN	-1.69	0	0
65255	*Asb4*	IPF-UP siAURKB-DN	-1.75	0	0
211134	*Lzts1*	IPF-UP siAURKB-DN	-1.76	0.01	0.03
14169	*Fgf14*	IPF-UP siAURKB-DN	-1.76	0.01	0.03
12124	*Bik*	IPF-UP siAURKB-DN	-1.76	0.01	0.02
66813	*Bcl2l14*	IPF-UP siAURKB-DN	-1.8	0	0
17022	*Lum*	IPF-UP siAURKB-DN	-1.81	0	0
223272	*Itgbl1*	IPF-UP siAURKB-DN	-1.82	0	0
22061	*Trp63*	IPF-UP siAURKB-DN	-1.97	0.01	0.02
67742	*Samsn1*	IPF-UP siAURKB-DN	-1.98	0	0
74186	*Ccdc3*	IPF-UP siAURKB-DN	-2.02	0	0
210622	*Pamr1*	IPF-UP siAURKB-DN	-2.02	0	0
228543	*Rhov*	IPF-UP siAURKB-DN	-2.12	0.01	0.02
11830	*Aqp5*	IPF-UP siAURKB-DN	-2.18	0	0
71693	*Colec11*	IPF-UP siAURKB-DN	-2.35	0	0
21380	*Tbx1*	IPF-UP siAURKB-DN	-2.43	0.01	0.02
18155	*Pnoc*	IPF-UP siAURKB-DN	-2.47	0	0
69908	*Rab3b*	IPF-UP siAURKB-DN	-2.8	0	0

Appendix Table S2. The list of human RT-PCR primers used in the study.

Gene symbol	Forward	Reverse
β-actin	CCAACCGCGAGAAGATGA	CCAGAGGCGTACAGGGATAG
AURKB	GATGGCCCAGAAGGAGAACT	AGGCTCTTTCCGGAGGACT
BAK	CCTGCCCTCTGCTTCTGA	CTGCTGATGGCGGTAAAAA
BAX	AGCAAACTGGTGCTCAAGG	CTTGGATCCAGCCCAAC
CCNA2	GGTACTGAAGTCCGGGAACC	GAAGATCCTTAAGGGGTGCAA
FAS	GTGGACCCGCTCAGTACG	TCTAGCAACAGACGTAAGAACCA
PLK1	AACGACTTCGTGTTCGTGGT	AGGGCTTTCCTCCTCTTGTG
WT1	AGCTGTCCCACTTACAGATGC	CCTTGAAGTCACACTGGTATGG

Appendix Table S3. The list of mouse RT-PCR primers used in the study.

Gene	Forward	Reverse
Hprt	GCCCTTGACTATAATGAGTACTTC AGG	TTCAACTTGCGCTCATCTTAGG
Aurka	GGGACATGGCTGTTGAGG	GTTTTCTTTACATCTGTCCATGTCA
Aurkb	ATTGCAGACTTTGGCTGGTC	AATCATCTCTGGGGGCAGAT
Bak	GGAATGCCTACGAACTCTTCA	CCAGCTGATGCCACTCTTAAA
Bax	GTGAGCGGCTGCTTGTCT	GGTCCCGAAGTAGGAGAGGA
Col1α1	AGACATGTTCAGCTTTGTGGAC	GCAGCTGACTTCAGGGATG
Col3α	CTCCTGGTGAGCGAGGAC	GACCAGGTTGCCCATCACT
Col5α	CTACATCCGTGCCCTGGT	CCAGCACCGTCTTCTGGTAG
Col14	ACGACGTGACTGAGAACAGC	AATGTCTGTGTGTGTCTCTCCAA
Col15	CCAGGGCTAAAAGGAGAACA	GGACGTCCCCAGTCAAGA
Fn1	CGGAGAGAGTGCCCCTACTA	CGATATTGGTGAATCGCAGA
Fas	AAACCAGACTTCTACTGCGATTCT	GGGTTCCATGTTCACACGA
CcnA2	CTTGGCTGCACCAACAGTAA	CAAACTCAGTTCTCCCAAAAACA
Ccnb1	GCGCTGAAAATTCTTGACAAC	TTCTTAGCCAGGTGCTGCAT

Plk1	TTGTAGTTTTGGAGCTCTGTCG	CAGTGCCTTCCTCCTCTTGT
Wt1	CAG ATG AAC CTA GGA GCT ACC TTA AA	TGC CCT TCT GTC CAT TTC A

Appendix Table S4. Antibodies and their dilutions used for Immunostaining.

Antibody	Dilution	Catalogue#	Company
Aurkb	1:600	Ab2254	Abcam
Vimentin	1:100	Sc-7557	Santa Cruz Biotechnology
PCNA	1:2000	2586	Cell Signaling Technology
Ki-67	1:100	556003	BD Biosciences
Ki-67	1:600	12202	Cell Signaling Technology
αSMA	1:2000	A5228	Sigma

Appendix Table S5. Antibodies and their dilutions used for immunoblotting**.**

Antibody	Dilution	Catalogue#	Company
Aurka	1:500	Ab13824	Abcam
Aurkb	1:1000	3094S	Cell Signaling Technology
WT1	1:500	12609-1-AP	Proteintech
PCNA	1:1000	2586S	Cell Signaling Technology
Col1	1:300	Sc-25974	Santa Cruz Biotechnology
Fn1	1:500	SC-9068	Santa Cruz Biotechnology
GAPDH	1:2000	A300-641	Bethyl Laboratories
αSma	1:20,000	A2547	Sigma

Appendix Table S6. P values and statistical test used to compare between groups in figures.

Figure 1	P value	Comparison	Statistical test
1A	<0.0001	Media vs TGFα	One-way ANOVA
	0.0152	Media vs CTGF	One-way ANOVA
	0.0005	Media vs IGF1	One-way ANOVA
1B	0.0028	Normal vs IPF	Unpaired t test
1D	0.0223	Aurkb: Normal lung vs Fibrotic lung	Unpaired t test
	0.5492	Aurka: Normal lung vs Fibrotic lung	Unpaired t test

1F	0.0025	Saline vs Bleomycin	Unpaired t test
**Figure 2**	**P value**	**Comparison**	**Statistical test**
2A	<0.0001	siControl vs siWT1	Unpaired t test
2B	<0.0001	siControl vs siWt1	Unpaired t test
2C	0.0097	Ad-Control vs Ad-WT1	Unpaired t test
2D	<0.0001	Media: Sc siRNA vs WT1 siRNA	One-way ANOVA
	<0.0001	Media vs TGFα: Sc siRNA vs Sc siRNA	One-way ANOVA
	<0.0001	TGFα: Sc siRNA vs WT1 siRNA	One-way ANOVA
2E	0.0004	IgG ctrl vs Anti-Wt1	Unpaired t test
2F	<0.0001	Con vs WT1$_{OE}$: Emp-Luc vs AURKB-Luc	One-way ANOVA
	<0.0001	Con vs WT1$_{OE}$: AURKB-Luc vs AURKB-Luc	One-way ANOVA
	<0.0001	WT1$_{OE}$ vs WT1$_{OE}$: Emp-Luc vs AURKB-Luc	One-way ANOVA
**Figure 3**	**P value**	**Comparison**	**Statistical test**
3D	<0.0001	Normal vs Fibrosis	Unpaired t test
3E	<0.0001	siControl vs siAURKB	Unpaired t test
	<0.0001	siControl vs siAurkb	Unpaired t test
3F	<0.0001	Media vs TGFα (siControl)	One-way ANOVA
	<0.0001	siControl vs siAurkb (TGFα)	One-way ANOVA
3G	0.0013	CCNA2: siControl vs siAURKB	Unpaired t test
	0.0002	PLK1: siControl vs siAURKB	Unpaired t test
3I	<0.0001	siControl vs siAURKB	2way ANOVA
	<0.0001	siControl vs siAurkb	2way ANOVA
3J	0.0032	BAK: siControl vs siAURKB	Unpaired t test
	0.0007	BAX: siControl vs siAURKB	Unpaired t test
	0.0003	FAS: siControl vs siAURKB	Unpaired t test
3K	0.0341	Bak: siControl vs siAurkb	Unpaired t test
	0.0488	Bax: siControl vs siAurkb	Unpaired t test
	0.0087	Fas: siControl vs siAurkb	Unpaired t test
**Figure 4**	**P value**	**Comparison**	**Statistical test**
4A	<0.0001	Vehicle vs Barasertib (1µM)	One way ANOVA
	<0.0001	Vehicle vs Barasertib (2µM)	One way ANOVA
	<0.0001	Vehicle vs Barasertib (5µM)	One way ANOVA
4B	0.0001	Vehicle vs Barasertib (5µM)	One way ANOVA
4C	<0.0001	Vehicle vs Barasertib	Unpaired t test
4D	0.0018	TGFα vs TGFα+Barasertib (5µM)	One way ANOVA

4E	<0.0001	Vehicle vs Barasertib	2Way ANOVA
**Figure 5**	**P value**	**Comparison**	**Statistical test**
5C	<0.0001	Control+Vehicle vs TGFα+Vehicle	One way ANOVA
	0.0006	TGFα+Vehicle vs TGFα+Barasertib	One way ANOVA
5D	0.0002	Control+Vehicle vs TGFα+Vehicle	One way ANOVA
	0.0222	TGFα+Vehicle vs TGFα+Barasertib	One way ANOVA
5E	<0.0001	Col1α1: Control+Veh vs TGFα+Veh	One way ANOVA
	<0.0001	Col1α1:TGFα+Veh vs TGFα+Barasertib	One way ANOVA
	<0.0001	Col5α:Control+Veh vs TGFα+Veh	One way ANOVA
	0.0004	Col5α:TGFα+Veh vs TGFα+Barasertib	One way ANOVA
	<0.0001	Fn1:Control+Veh vs TGFα+Veh	One way ANOVA
	<0.0001	Fn1:TGFα+Veh vs TGFα+Barasertib	One way ANOVA
**Figure 6**	**P value**	**Comparison**	**Statistical test**
6B	0.0018	Normal+Vehicle vs TGFα+Vehicle	One way ANOVA
	0.0011	TGFα+Vehicle vs TGFα+Barasertib	One way ANOVA
6C	<0.0001	Aurkb: Control+Veh vs TGFα+Veh	One way ANOVA
	0.0001	Aurkb:TGFα+Veh vs TGFα+Barasertib	One way ANOVA
	0.0046	CcnA2:Control+Veh vs TGFα+Veh	One way ANOVA
	0.0052	CcnA2:TGFα+Veh vs TGFα+Barasertib	One way ANOVA
	<0.0001	Plk1:Control+Veh vs TGFα+Veh	One way ANOVA
	0.0093	Plk1:TGFα+Veh vs TGFα+Barasertib	One way ANOVA
**Figure 7**	**P value**	**Comparison**	**Statistical test**
7B	<0.0001	Control+Vehicle vs TGFα+Vehicle	One way ANOVA
	0.0025	TGFα+Vehicle vs TGFα+Barasertib	One way ANOVA
7C	0.0116	Normal+Vehicle vs TGFα+Vehicle	One way ANOVA
	0.0041	TGFα+Vehicle vs TGFα+Barasertib	One way ANOVA
	0.0007	Normal+Vehicle vs TGFα+Vehicle	One way ANOVA
	0.0014	TGFα+Vehicle vs TGFα+Barasertib	One way ANOVA
7E	0.0010	Resistance: Normal+Vehicle vs TGFα+Vehicle	One way ANOVA
	0.0275	Resistance: TGFα+Vehicle vs TGFα+Barasertib	One way ANOVA
	0.0001	Elastance: Normal +Vehicle vs TGFα+Vehicle	One way ANOVA
	0.0130	Elastance: TGFα+Vehicle vs TGFα+Barasertib	One way ANOVA

	<0.0001	Compliance: Normal +Vehicle vs TGFα+Vehicle	One way ANOVA
	0.0205	Compliance : TGFα+Vehicle vs TGFα+Barasertib	One way ANOVA
**Figure 8**	**P value**	**Comparison**	**Statistical test**
8C	0.0008	Veh vs Veh+ Bleomycin	One way ANOVA
	0.0466	Veh vs Bleomycin +Barasertib	One way ANOVA
8D	0.0089	Col1α1: Vehicle vs Barasertib	Unpaired t test
	0.0027	Col3α: Vehicle vs Barasertib	Unpaired t test
	0.0025	Col5α : Vehicle vs Barasertib	Unpaired t test
	0.0004	Col14: Vehicle vs Barasertib	Unpaired t test
	<0.0001	Col15: Vehicle vs Barasertib	Unpaired t test
	0.0440	Ccna2: Vehicle vs Barasertib	Unpaired t test
	0.0017	Ccnb1: Vehicle vs Barasertib	Unpaired t test
	0.0197	Fas: Vehicle vs Barasertib	Unpaired t test
8E	0.0025	Vehicle vs Barasertib	Unpaired t test
**Figure EV2**	**P value**	**Comparison**	**Statistical test**
EV2A	0.0011	AdenoCtrl vs AdenoWT1	Unpaired t test
**Figure EV3**	**P value**	**Comparison**	**Statistical test**
EV3A	<0.0001	siControl vs siAurkb	Unpaired t test
**Figure EV4**	**P value**	**Comparison**	**Statistical test**
EV4	0.0111	CCNA2: Vehicle vs Barasertib	Unpaired t test
	0.0421	PLK1: Vehicle vs Barasertib	Unpaired t test
**Figure EV5**	**P value**	**Comparison**	**Statistical test**
	<0.0001	Control +Vehicle vs TGFα+Vehicle	One way ANOVA
	0.0196	TGFα+Vehicle vs TGFα+Barasertib	One way ANOVA
