# Supplementary material for: Inhibition of Aurora Kinase B attenuates fibroblast activation and pulmonary fibrosis
Source: EMBO Mol Med. 2020 Aug 6;12(9):e12131. doi: 10.15252/emmm.202012131 (PMC7507328; doi:10.15252/emmm.202012131)
Supplement: Supplementary file 2 — Expanded View Figures PDF [file EMMM-12-e12131-s002.pdf]

Expanded View Figures

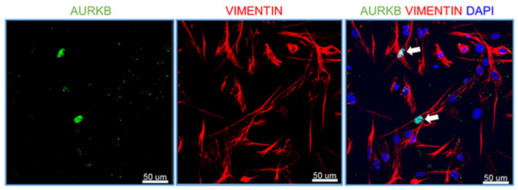

Figure EV1. Lung-resident fibroblasts show AURKB localized in the nucleus.

Primary lung-resident fibroblasts were isolated from lung fibroblast cultures of TGFα mice on Dox for 4 weeks. Co-immunostaining was performed using antibodies against AURKB (Green) and Vimentin (Red). All images were obtained at 40× magnification. Scale bar, 50 µm.

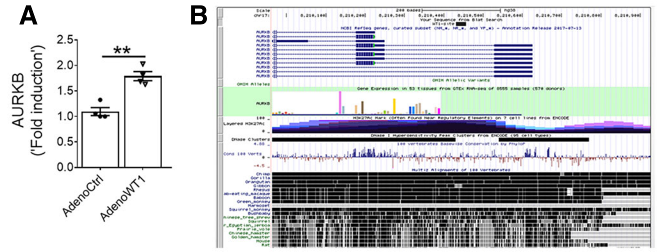

Figure EV2. WT1 increases AURKB expression.

A Quantification of AURKB transcripts in non-IPF fibroblasts transduced with control or WT1 adenoviral particles for 72 h ($n = 4$). Data were presented as mean ± SEM. **$P < 0.005$, unpaired *t*-test.

B Schematic illustration of AURKB gene with location of the putative WT1 binding sites that were conserved among mammals including humans and mice.

Data information: All data are presented as mean ± SEM.

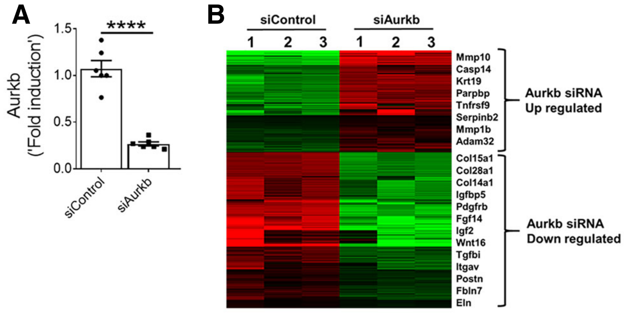

Figure EV3. The knockdown of AURKB alters IPF-specific gene network.

A Primary lung-resident fibroblasts isolated from TGFα mice on Dox for 10 days. Cells were transiently transfected with control or Aurkb siRNA, and transcripts were quantified by RT–PCR after 72 h. Data were presented as mean ± SEM. ****$P < 0.00005$, unpaired *t*-test ($n = 3$).

B Heat map shows two clusters of differentially expressed genes up- or downregulated (indicated with color key) by twofold or more upon genetic knockdown of Aurkb compared to control siRNA. ($n = 3$).

Data information: All data were presented as mean ± SEM.

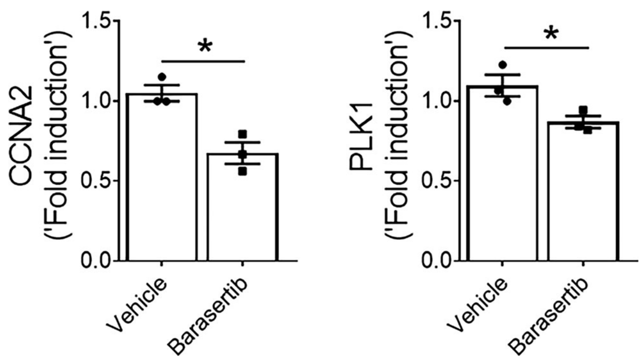

Figure EV4. Barasertib treatment attenuates the expression of fibroproliferative genes.

Quantification of CCNA2 and PLK1 transcripts in IPF fibroblasts treated with vehicle or barasertib (1 µM) for 16 h. Data were presented as mean ± SEM. ($n = 4$). *$P < 0.05$, unpaired *t*-test.

Data information: All data were presented as mean ± SEM.

© 2020 The Authors

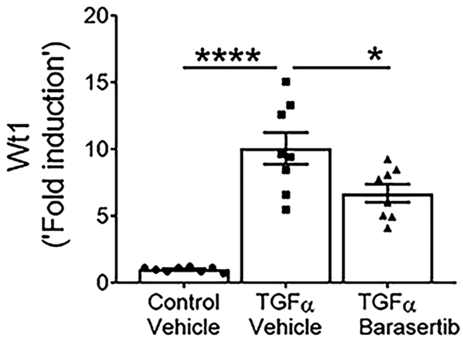

Figure EV5. Barasertib inhibits Wt1 expression in the lungs of TGFα mice.
Quantification of Wt1 gene transcripts in total lung transcripts of mice treated with vehicle or barasertib for 4 weeks. Data were presented as mean ± SEM. $^*P < 0.05$, $^{****}P < 0.00005$, 1-way ANOVA, ($n = 8$ mice/group).

 © 2020 The Authors
