# Supplementary material for: Inhibition of Aurora Kinase B attenuates fibroblast activation and pulmonary fibrosis
Source: EMBO Mol Med. 2020 Aug 6;12(9):e12131. doi: 10.15252/emmm.202012131 (PMC7507328; doi:10.15252/emmm.202012131)

Figure 1

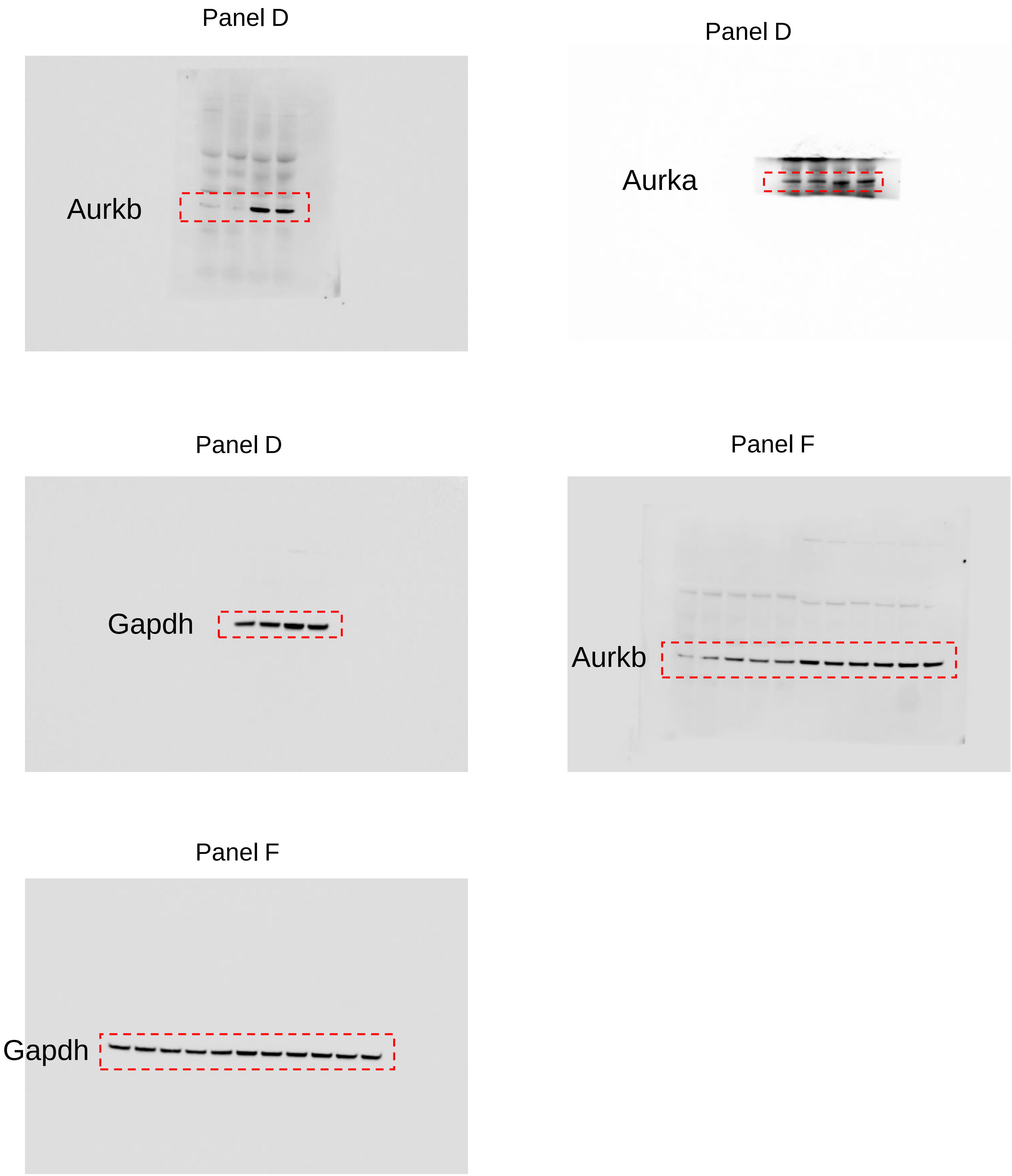
Panel D
Aurkb
Panel D
Aurka
Panel D
Gapdh
Panel F
Aurkb
Panel F
Gapdh

Figure 1

Panel D: Biological replicates

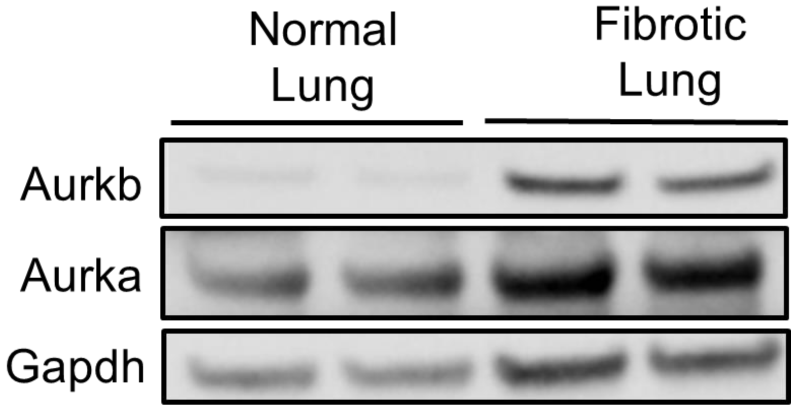

Panel C: Low magnification images

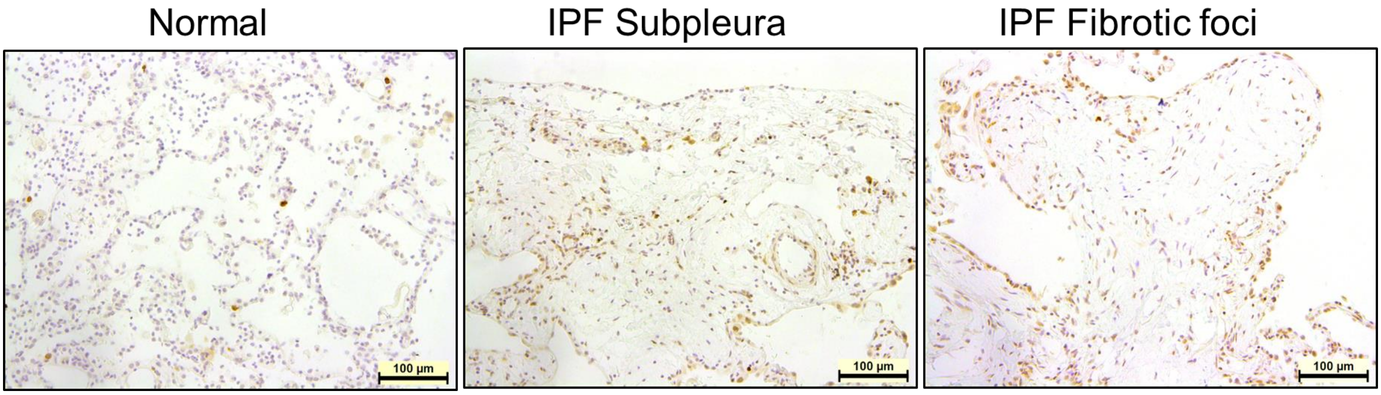

Panel C: Negative control

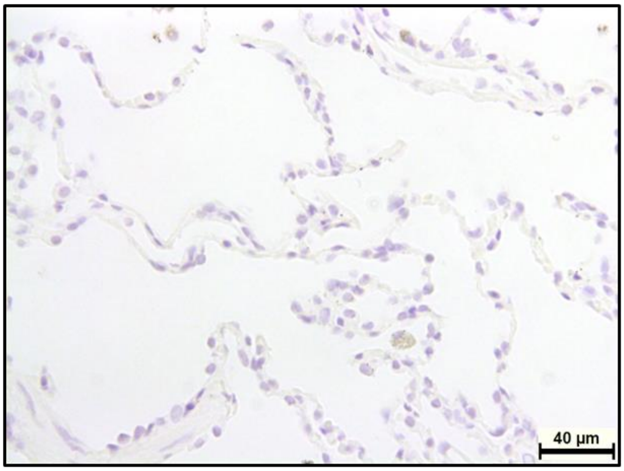

Panel E: Negative control

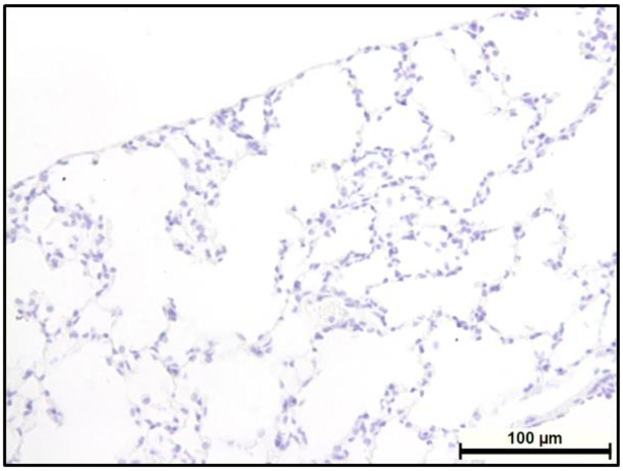

Supplement: Supplementary file 4 — Source Data for Figure 1 [file EMMM-12-e12131-s003.pdf]
