# Supplementary figures and images for: Inhibition of Aurora Kinase B attenuates fibroblast activation and pulmonary fibrosis
Source: EMBO Mol Med. 2020 Aug 6;12(9):e12131. doi: 10.15252/emmm.202012131 (PMC7507328; doi:10.15252/emmm.202012131)

Figure 2

Panel C

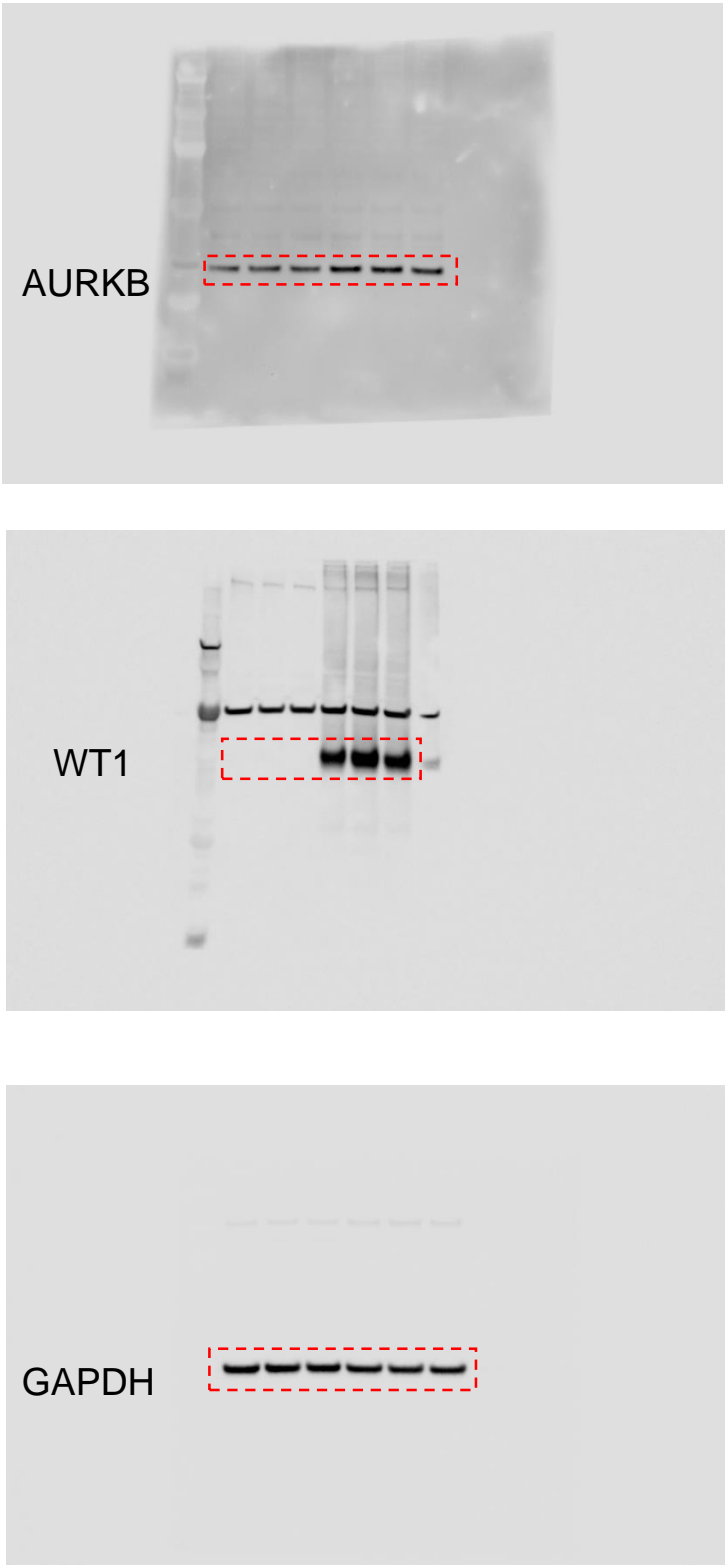

Supplement: Supplementary file 5 — Source Data for Figure 2 [file EMMM-12-e12131-s004.pdf]

**Figure 3**

Panel H: Low magnification images

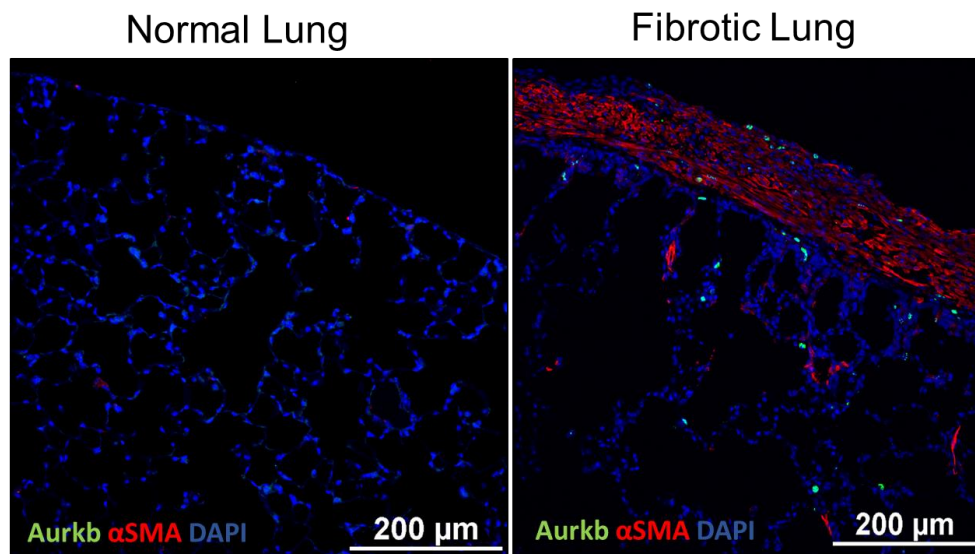

Panel C: Negative control

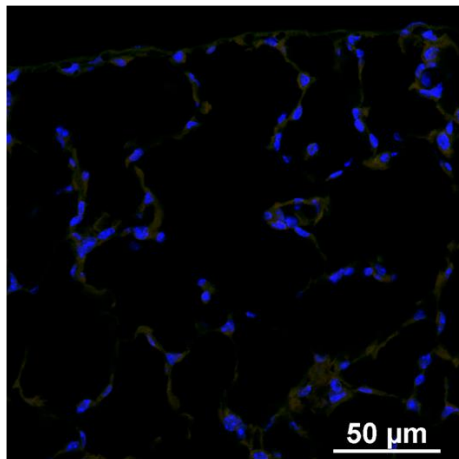

Panel H: Negative control

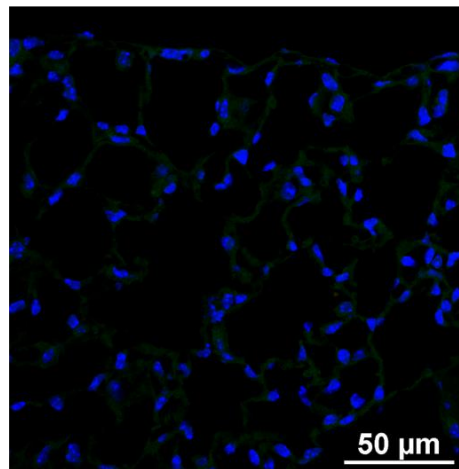

Supplement: Supplementary file 6 — Source Data for Figure 4 [file EMMM-12-e12131-s005.pdf]

Figure 6

Panel A: Low magnification images

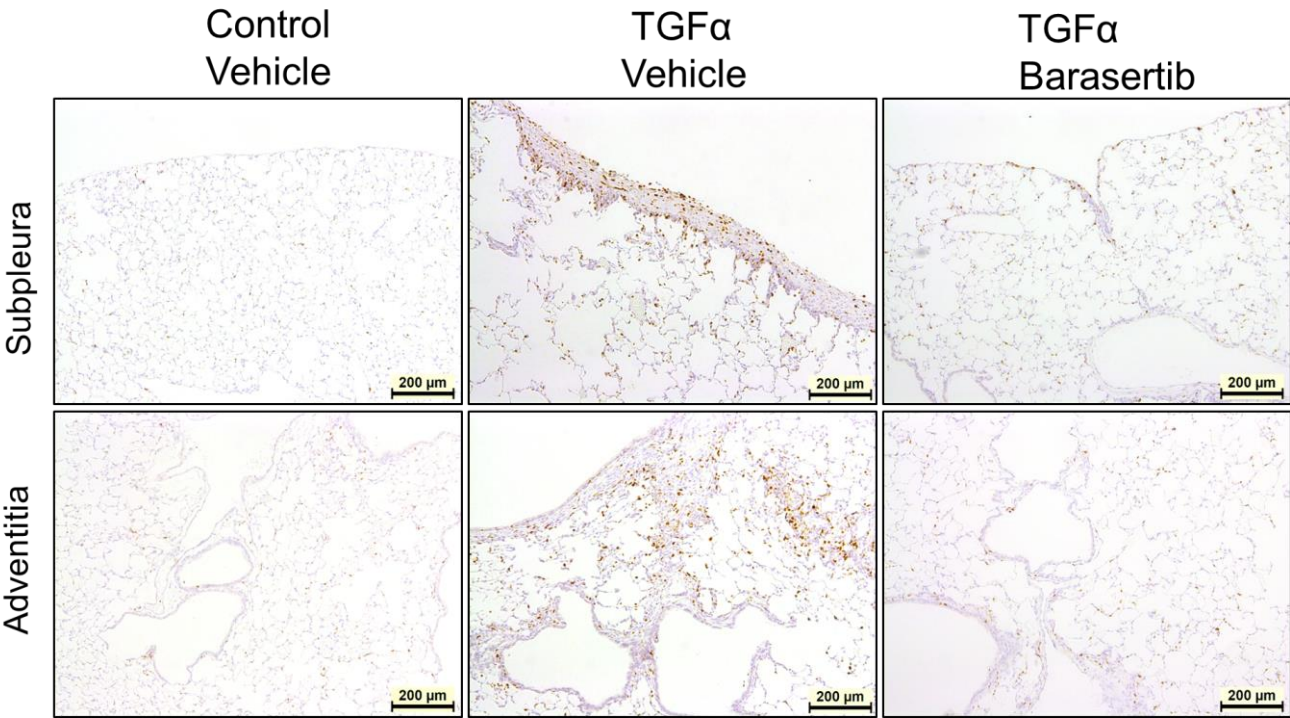

Panel A: Negative control

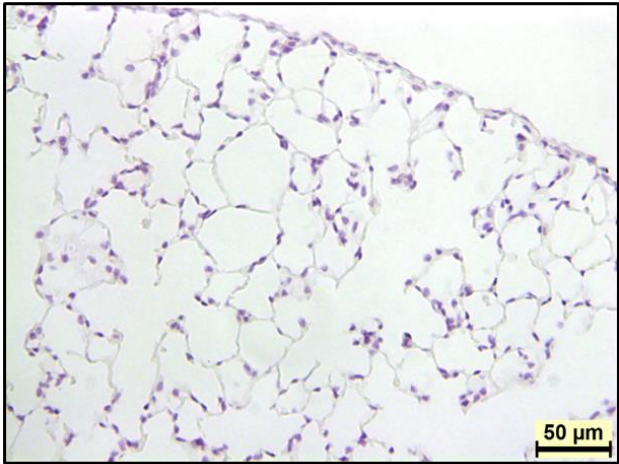

Panel B

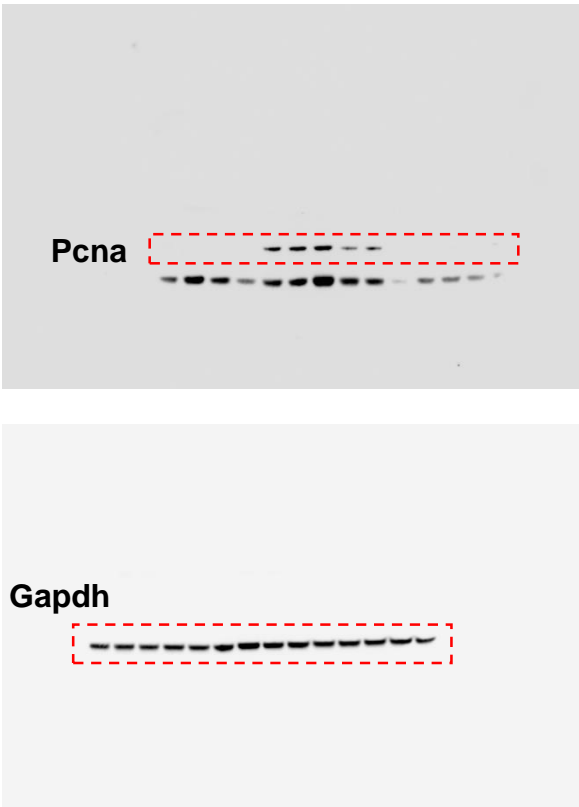

Supplement: Supplementary file 7 — Source Data for Figure 6 [file EMMM-12-e12131-s006.pdf]
